# Disposable non-enzymatic impedimetric biosensor using Mn-doped ZnS-chitosan nanocomposite for tetracycline detection

**DOI:** 10.1371/journal.pone.0344103

**Published:** 2026-02-27

**Authors:** Trong-Du Nguyen, Huy Quang Nguyen, Mai Thi Tran, Son Hai Nguyen, Cuong Danh Do

**Affiliations:** 1 School of Mechanical Engineering, Hanoi University of Science and Technology, Hanoi, Vietnam; 2 College of Engineering and Computer Science, VinUniversity, Hanoi, Vietnam; 3 Smart Green Transformation Center (Green-X), VinUniversity, Hanoi, Vietnam; Islamic Azad University, IRAN, ISLAMIC REPUBLIC OF

## Abstract

Monitoring antibiotic residues in aquaculture water is critical for food safety, environmental protection, and antimicrobial stewardship. Here, we present a proof-of-concept disposable, non-enzymatic impedimetric biosensor for the rapid and selective detection of tetracycline. The sensor employs interdigitated electrodes functionalized with a manganese-doped zinc sulfide-chitosan nanocomposite, providing a stable, conductive, and environmentally friendly sensing interface. The successful synthesis of the nanocomposite was confirmed using scanning electron microscopy, high-resolution transmission electron microscopy, X-ray diffraction, energy-dispersive X-ray spectroscopy, and Fourier transform infrared spectroscopy. Using electrochemical impedance spectroscopy, the device exhibits a linear response over the range of 62.5–1000 nM tetracycline, with a limit of detection of 42 nM and a limit of quantification of 138 nM. It also displays strong selectivity over other common antibiotics, including ampicillin, amoxicillin, cephalexin, doxycycline, penicillin, and non-antibiotic interferent, glucose, as well as excellent reproducibility and operational stability under repeated measurements. The sensor can detect tetracycline in lake, tap, and bottled water with linear responses across the same concentration range. The combination of biocompatible, low-cost materials and simple fabrication supports single-use deployment and scalability. These results demonstrate the potential of manganese-doped zinc sulfide-chitosan nanocomposite-based impedimetric biosensors as practical platforms for on-site monitoring of antibiotic residues in aquaculture water.

## Introduction

Tetracycline (TET) is a broad-spectrum antibiotic widely used in both human healthcare and veterinary medicine. In aquaculture, it is among the most commonly applied antibiotics for disease control, prophylaxis, and growth promotion owing to its high efficacy and low cost [1,2]. However, unregulated and excessive use in fish and shrimp farming has led to the accumulation of tetracycline residues in aquaculture water, cultured organisms, and downstream environments such as rivers and sediments [3,4]. These residues pose serious risks to aquatic ecosystems and food safety and accelerate the development and spread of antimicrobial resistance, a major global public health concern [5]. Continuous monitoring of tetracycline in aquaculture systems is therefore essential to ensure compliance with food safety regulations and to promote sustainable aquaculture practices.

A variety of analytical techniques, including high-performance liquid chromatography, liquid chromatography–tandem mass spectrometry, and enzyme-linked immunosorbent assays, have been developed to detect tetracycline residues in food and environmental samples [6–8]. Although these methods offer high accuracy and sensitivity, their routine implementation in aquaculture facilities is limited by the need for sophisticated instrumentation, laborious sample preparation, and skilled personnel. These drawbacks make them unsuitable for rapid, on-site monitoring, where timely detection is critical for maintaining safe antibiotic levels in aquaculture water and products.

Electrochemical biosensors have emerged as promising alternatives to conventional methods for antibiotic detection because of their simplicity, low cost, and ability to deliver rapid, sensitive measurements [9,10]. Among electrochemical approaches, non-enzymatic impedimetric biosensors based on electrochemical impedance spectroscopy (EIS) are particularly attractive, as they enable label-free detection compatible with real-time measurements and can be integrated into miniaturized, portable devices. These features make them well-suited to in situ aquaculture monitoring, where non-destructive, continuous assessment of water quality is needed to mitigate antibiotic overuse and contamination. EIS sensors employing interdigitated electrodes (IDEs) have further advanced this field due to their ease of fabrication, low power consumption, and high signal-to-noise ratio [11,12].

Biopolymer-based nanocomposites have gained increasing attention as cost-effective, sustainable, and biocompatible sensing materials. Manganese-doped zinc sulfide (Mn:ZnS) nanoparticles exhibit tunable optical and electrical properties, high stability, and low toxicity [13]. When embedded in a chitosan matrix-a biodegradable, film-forming natural polymer derived from crustacean shells, the resulting Mn:ZnS-chitosan (Mn:ZnS-CH) nanocomposite provides a stable, conductive, and environmentally friendly sensing interface. Previous studies have primarily applied Mn:ZnS-CH to fluorescence-based antibiotic detection [14–17], whereas its potential in non-enzymatic impedimetric biosensing for aquaculture applications remains largely unexplored.

Here, we present a disposable, non-enzymatic impedimetric biosensor based on electrochemical impedance spectroscopy that employs interdigitated electrodes modified with Mn:ZnS-CH nanocomposite for the selective detection of tetracycline in aquaculture water. The sensor exhibits high sensitivity over the concentration range 62.5–1000 nM, excellent selectivity against structurally related antibiotics (amoxicillin, cephalexin, ampicillin, doxycycline, and penicillin), and non-antibiotic interference, glucose, and strong reproducibility. To the best of our knowledge, this is the first report to employ Mn:ZnS-CH nanocomposite in a non-enzymatic impedimetric configuration using IDEs for antibiotic detection. While our previous work explored the optical (fluorescence- and absorbance-based) response of Mn:ZnS-CH materials [15–17], the present study introduces an electrical transduction mechanism governed by changes in interfacial charge-transfer resistance within the biopolymer matrix. This shift from optical to impedimetric sensing enables label-free, real-time quantification and supports a low-cost, disposable architecture tailored for on-site monitoring in aquaculture environments.

## Methods

### Disposable impedance biosensor fabrication

All chemicals, antibiotics, and the synthesis procedures for Mn:ZnS-CH nanocomposite have been described in detail previously [18]. In brief, Mn:ZnS-chitosan nanocomposites were prepared using a precipitation-assisted coating approach. Zinc acetate dihydrate and manganese chloride tetrahydrate were dissolved in deionized water, whereas chitosan was dissolved in dilute acetic acid and added dropwise to the metal precursor solution under continuous stirring. Chitosan (CAS No. 9012-76-4) was obtained from Shanghai Zhanyun Chemical Co., Ltd. According to the supplier, the material exhibits a degree of deacetylation of at least 90% and a molecular weight of approximately 100–400 kDa. This high degree of deacetylation increases the density of free amino groups, enhancing solubility in acidic media and facilitating interactions with target analytes or subsequent chemical modification [19]. Sodium sulfide nonahydrate was then introduced as the sulfur source, and the resulting suspension was heated at 80 °C for 2 h in a sealed vessel. The product was collected by centrifugation, washed repeatedly with ethanol and deionized water, and dried at 60 °C to obtain chitosan-coated ZnS:Mn nanoparticles. For this study, antibiotic solutions were prepared at five concentrations: 62.5, 125, 250, 500, and 1000 nM.

**Fig 1:**
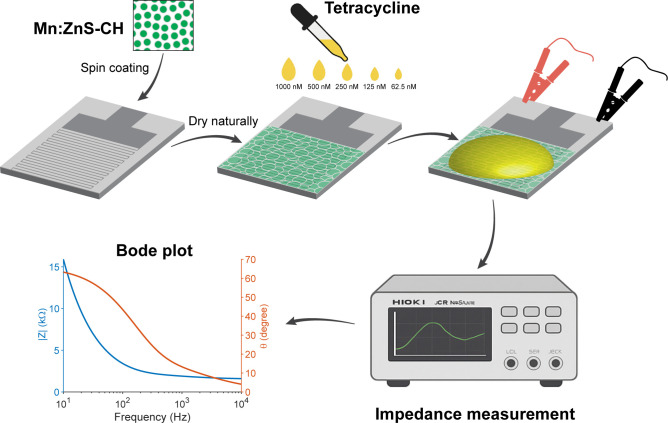
The schematic of the proposed biosensor’s fabrication.

The electrode design was adapted from previous studies [12,20] and shown in S1 Fig. To prepare a biosensor using Mn:ZnS-CH, clean electrodes were coated with 100 µL of a 5 mg/mL sensing material in two stages using a spin coater: first at 200 rpm for 10 seconds, followed by 1500 rpm for 10 seconds. The Mn:ZnS-CH layer forms a uniform, conductive biocoating without the need for additional binders or functionalization steps, thereby simplifying scale-up and supporting single-use deployment for field testing. The sensors were then tested with varying concentrations of tetracycline as shown in Fig 1. Electrochemical impedance measurements were carried out using a Hioki LCR IM3536 with an excitation voltage of 10 mV over a frequency range of 4–10^4^ Hz. All sensing performance experiments were conducted under ambient laboratory conditions at a temperature of 25 °C and 90% relative humidity.

### Data analysis using the equivalent Randles circuit

To evaluate the electrochemical behavior of the proposed biosensors, a defined volume of analyte solution was carefully dispensed onto the sensor surface. Electrochemical impedance spectroscopy measurements were then performed by applying a frequency range of 4 Hz to 10^4^ Hz. At each frequency and analyte concentration, both the impedance (|Z|) and phase angle (θ) were recorded.

The complex impedance data were converted into their real (Z_re_) and imaginary (Z_im_) components, defined as Z_re_ = |Z|cosθ, Z_im_ = |Z|sinθ, and Nyquist plots were constructed by plotting −Z_im_ versus Z_re_. These plots typically reveal the characteristic semicircular shape associated with charge transfer processes at the sensor-electrolyte interface.

**Fig 2:**
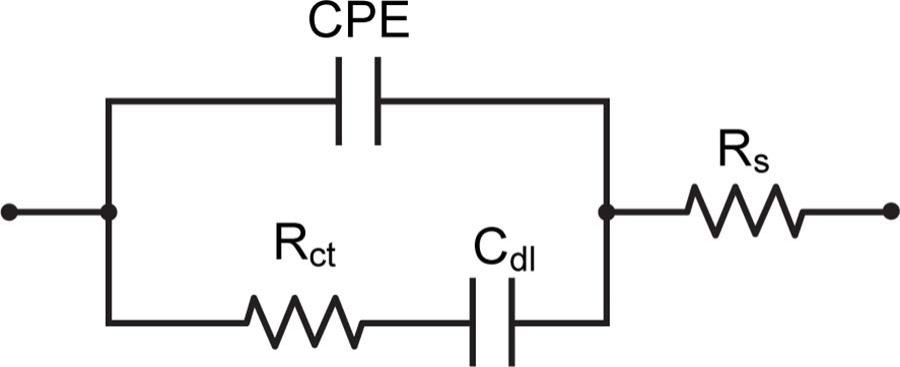
An equivalent Randles circuit model was used to fit the impedance spectra of the Mn:ZnS-CH/IDE biosensor in detecting tetracycline.

The impedance spectra were analyzed and fitted using ZView software (AMETEK Scientific Instruments). As shown in Fig 2, the data were modeled using an equivalent circuit consisting of a solution resistance (R_s_) in series with an interfacial branch comprising the charge-transfer resistance (R_ct_), the double-layer capacitance (C_dl_), and a constant-phase element (CPE). This circuit accounts for non-ideal capacitive behavior arising from surface roughness, heterogeneity, and distributed interfacial time constants at the Mn:ZnS-CH-modified interdigitated electrodes. The selection of this model was guided by the observation that the Nyquist plots are dominated by a single semicircle over the investigated frequency range, indicating that interfacial charge-transfer processes govern the impedance response. However, the slight depression of the semicircle suggests a non-ideal capacitive element is required for accurate representation. After evaluating alternative equivalent circuits, this model was selected as it provided the most robust and reproducible fitting performance with minimal fitting error, while preserving clear discrimination of R_ct_ responses among different analytes. No pronounced low-frequency diffusion tail was observed; therefore, a Warburg diffusion element was not included in the fitting model.

The impedance of the CPE is described by: Z_CPE_ = 1/C(jω)^n^, where j is the imaginary unit, ω is the angular frequency, C is the pseudo-capacitance, and n (0.5 < n < 1) represents the deviation from ideal capacitive behavior.

Among the fitted parameters, the charge-transfer resistance R_ct_ is the primary indicator of sensing performance, as it reflects the resistance to electron transfer at the electrode-electrolyte interface. A decrease in R_ct_ corresponds to enhanced charge transfer, typically induced by the binding of tetracycline molecules to the Mn:ZnS-CH layer. Changes in C_dl_ and CPE provide additional insight into the interfacial structure and molecular interactions at the electrode surface.

## Results and discussion

### Materials characterization

#### Morphology analysis.

**Fig 3:**
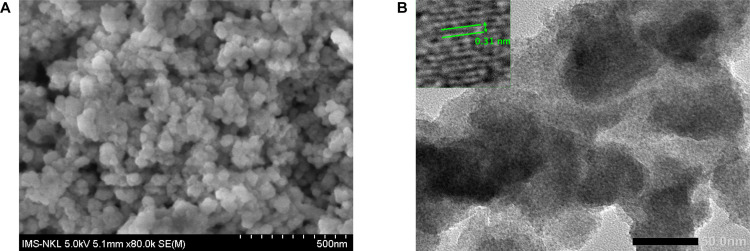
Morphology analysis. **(A)** SEM image acquired using a HITACHI S-4800 system (Hitachi High-Tech, Japan) and **(B)** HRTEM image captured using a JEM 2100 microscope (JEOL Ltd., Japan) of the prepared materials.

The morphology and microstructural features of the prepared Mn:ZnS-CH material were investigated using scanning electron microscopy (SEM) and high-resolution transmission electron microscopy (HRTEM), as shown in Fig 3. The SEM image (Fig 3A) reveals that the material is composed of densely packed nanoparticles with relatively uniform size distribution and without obvious large-scale agglomeration, indicating good dispersity of the synthesized nanocomposite. The granular surface morphology suggests effective nucleation and growth of ZnS-based nanostructures within the chitosan matrix.

Further insights into the internal structure were obtained from HRTEM analysis (Fig 3B), which confirms the formation of nanoscale particles with well-defined boundaries and relatively homogeneous contrast, consistent with the SEM observations. The average particle size is estimated to be on the order of several tens of nanometers. In addition, the high-magnification HRTEM image (Fig 3B) clearly shows well-resolved lattice fringes with an interplanar spacing of approximately 0.31 nm, which can be assigned to the characteristic crystallographic planes of crystalline ZnS. This observation confirms the successful formation of ZnS nanocrystals within the composite material, in agreement with previous reports [21].

**Fig 4:**
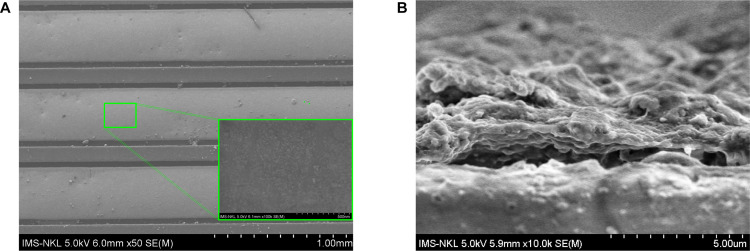
SEM images acquired using a HITACHI S-4800 system (Hitachi High-Tech, Japan) of the coated interdigitated electrodes. (A) top-view image of the electrodes, with a higher-magnification zoom-in of a selected region on a representative single electrode finger shown in the lower corner; (B) side-view (cross-sectional) image of the coated electrodes.

Fig 4 presents SEM images of the Mn:ZnS-CH -coated interdigitated electrodes, providing direct evidence of coating uniformity on the device level. As shown in the top-view SEM image (Fig 4A), the Mn:ZnS-CH layer continuously covers the electrode fingers and the interdigitated gaps without visible cracking, delamination, or large-scale agglomeration. The surface appears morphologically consistent along the electrode fingers, and the higher-magnification zoom-in image of a representative single finger (inset in Fig 4A) reveals a relatively homogeneous coating without pronounced pinholes or local defects. These observations indicate that the Mn:ZnS-CH composite can form a uniform film on both the metallic electrodes and the adjacent insulating regions.

Further structural insight is provided by the side-view (cross-sectional) SEM image shown in Fig 4B. The cross-section confirms the formation of a continuous and conformal Mn:ZnS-CH layer along the electrode surface and across the finger edges, despite the step-height geometry inherent to the interdigitated electrode design. The coating thickness is estimated to be on the order of a few micrometers, with only limited local variation along the electrode profile. Although minor thickness fluctuations are expected for solution-processed composite films, the observed continuity and conformal coverage demonstrate sufficient lateral and vertical uniformity, which is essential for minimizing electrode-to-electrode variability and ensuring reproducible electrochemical sensing performance.

#### Structural characterizations.

**Fig 5:**
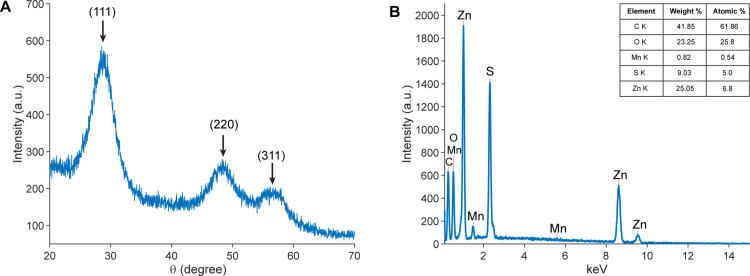
Structural characterizations. **(A)** XRD patterns acquired using a Rigaku MiniFlex 600 diffractometer (Rigaku Europe SE, Germany) and **(B)** EDX spectrum obtained using a HITACHI S-4800 system (Hitachi High-Tech, Japan) for the prepared materials.

The crystalline structure of the prepared materials was analyzed using X-ray diffraction (XRD), and the corresponding pattern is presented in Fig 5A. The diffraction peaks located at approximately 2θ ≈ 28.5°, 47.5°, and 56.3° can be indexed to the (111), (220), and (311) crystal planes of cubic ZnS, respectively (JCPDS card No. 5–0566) [22]. No additional peaks related to Mn-based secondary phases are observed within the detection limit of XRD, indicating that Mn incorporation does not alter the host ZnS crystal structure and occurs at a low doping level. The broad diffraction features further suggest nanoscale crystallite dimensions, consistent with HRTEM observations.

The elemental composition was examined by energy-dispersive X-ray spectroscopy (EDX) (Fig 5B), which confirmed the presence of Zn and S, along with a detectable Mn signal. Semi-quantitative EDX analysis estimates the Mn content at ~0.5 at.%, corresponding to low-level Mn doping. Consistent elemental signatures obtained from representative regions indicate a reasonably uniform Mn distribution at the microscale. Considering the intrinsic limitations of EDX, the incorporation of Mn into the ZnS lattice is supported by the combined evidence from XRD and EDX analyses.

#### Fourier transform infrared spectroscopy.

**Fig 6:**
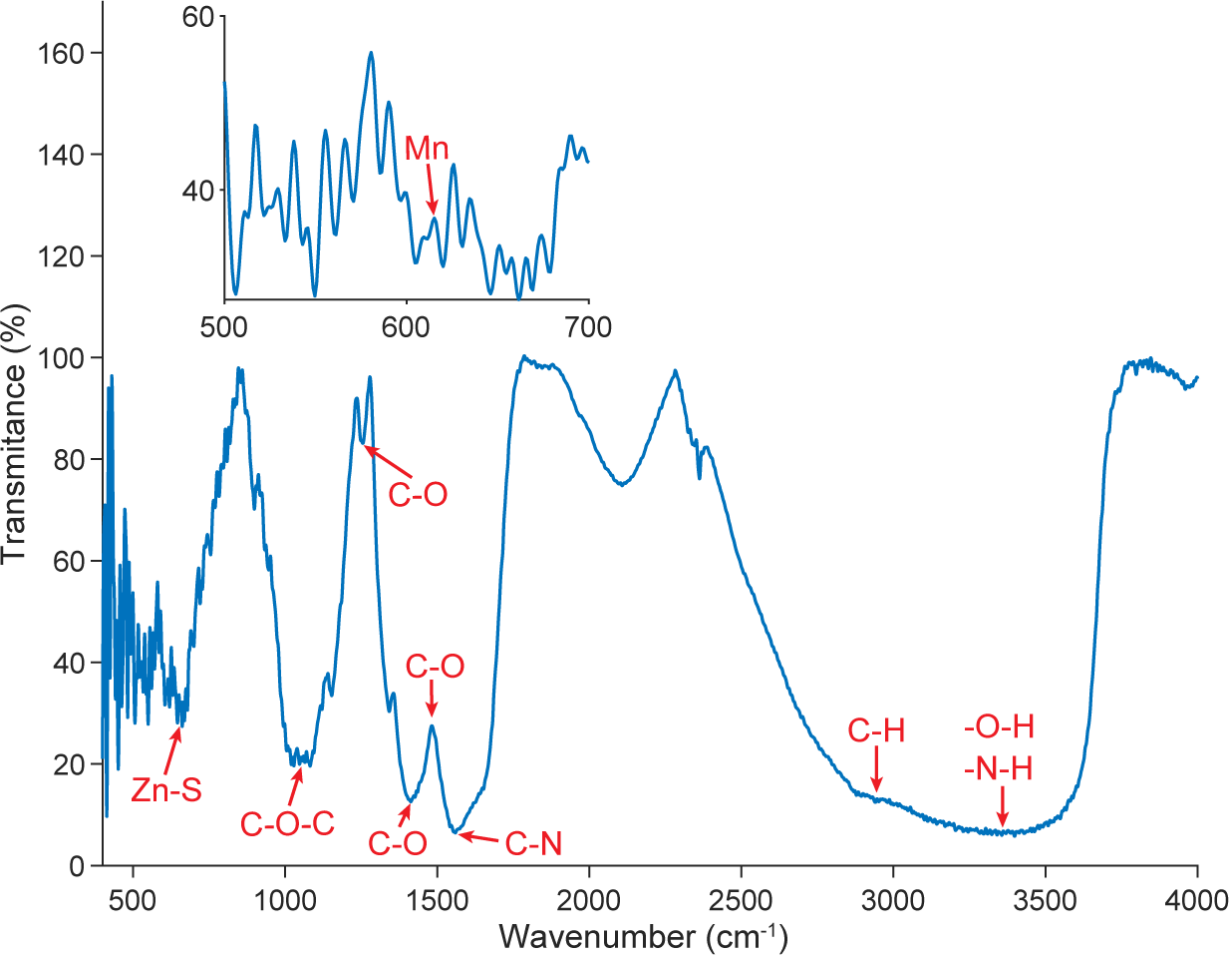
FTIR spectrum of the prepared materials acquired using a Jasco FTIR-4600 spectrometer (Jasco, Japan).

Fourier transform infrared (FTIR) spectroscopy was employed to examine the surface functional groups and chemical features of the prepared materials, and the corresponding spectra are shown in Fig 6. A broad absorption band observed in the region of approximately 3200−3500 cm^-1^ is attributed to the stretching vibrations of -OH and -NH groups, indicating the presence of hydroxyl and amine functionalities on the material surface. The absorption band around 2920 cm^-1^ corresponds to C-H stretching vibrations.

Characteristic absorption bands in the range of approximately 1000−1650 cm^-1^ are associated with C-O, C-N, and amide-related vibrations, suggesting interactions between the inorganic ZnS nanoparticles and the organic matrix. The vibrational modes characteristic of chitosan are identified by the N-H bending vibration at 3389 cm^-1^, the C-O stretching bands at 1412 and 1557 cm^-1^, and the C-O-C stretching vibration at 1047 cm^-1^, which are consistent with previously reported studies [23].

In addition, absorption peaks associated with ZnS are observed at 508, 616, and 1047 cm^-1^, while the presence of Mn is indicated by the band at 661 cm^-1^ and the splitting observed near 1047 cm^- 1^, in agreement with earlier reports on Mn-doped ZnS systems [24]. These spectral features collectively confirm the coexistence of ZnS nanocrystals and the chitosan-based matrix within the prepared materials.

### Tetracycline detection based on the charge transfer resistance

**Fig 7:**
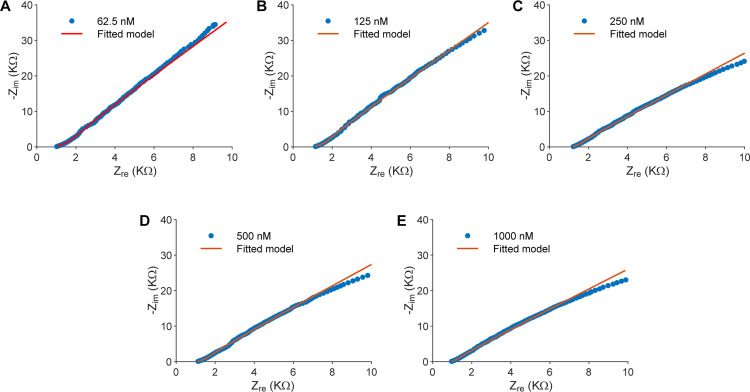
Nyquist plots of Mn:ZnS-CH-based biosensors in deionized water at different tetracycline concentrations. (A) 62.5 nM, (B) 125 nM, (C) 250 nM, (D) 500 nM, and (E) 1000 nM. The blue symbols represent the experimental data, and the red curves are the fitted responses obtained using the Randles equivalent circuit model shown in Fig 2.

Fig 7 shows the electrochemical impedance response of the biosensor to tetracycline in deionized (DI) water over the concentration range 62.5–1000 nM. A clear trend across all Nyquist plots is the systematic decrease in semicircle diameter with increasing tetracycline concentration. Because the semicircle diameter is primarily governed by the charge-transfer resistance R_ct_, this trend reflects a progressive reduction in R_ct_ as more tetracycline molecules interact with the sensing layer. The corresponding enhancement of electron-transfer kinetics at the electrode interface is consistent with increased analyte binding, which lowers the effective barrier for charge-transfer processes.

S1 Table summarizes the electrochemical parameters extracted using the Randles equivalent circuit model shown in Fig 2 of one representative biosensor, including the solution resistance R_s_, double-layer capacitance C_dl_, constant phase element CPE parameters, and charge-transfer resistance R_ct_. Among these parameters, R_ct_ exhibits a clear, monotonic, and concentration-dependent decrease with increasing tetracycline concentration, whereas the CPE magnitude and exponent (n) display comparatively larger variations. These variations are attributed to secondary interfacial effects rather than to the primary sensing mechanism.

The CPE accounts for non-ideal capacitive behavior arising from surface heterogeneity, polymer swelling, interfacial roughness, and local dielectric inhomogeneity at the Mn:ZnS-CH/electrolyte interface. The interaction between TET molecules and the chitosan matrix can modify hydration, local permittivity, and charge distribution within the polymer layer, resulting in observable changes in the CPE magnitude and exponent (n). Such behavior is characteristic of polymer-modified electrochemical interfaces and does not necessarily indicate a dominant kinetic or diffusion-controlled process.

Importantly, the observed variability in CPE parameters does not affect the interpretation of R_ct_ as the dominant analytical signal. Across all tested concentrations, R_ct_ maintains a robust, highly linear response with low fitting error, whereas the CPE parameters exhibit greater scatter and lower reproducibility. Consequently, CPE parameters are not suitable as primary analytical metrics, as they are influenced by multiple coupled physical factors that are not uniquely associated with TET binding. Accordingly, the equivalent circuit was intentionally selected to emphasize R_ct_ as the most physically meaningful and analytically reliable parameter, while variations in CPE are treated as complementary indicators of interfacial heterogeneity rather than confounding factors.

**Fig 8:**
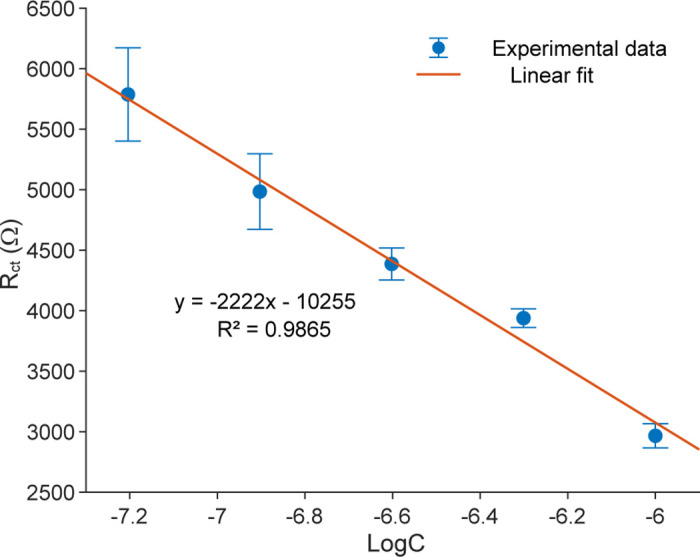
Charge transfer resistance R_ct_ of the biosensor as a function of the logarithm of tetracycline concentration, showing a linear calibration relationship.


As R_ct_ is the primary sensing parameter of the proposed biosensor, Table 1 summarizes the average R_ct_ values and the corresponding ΔR_ct_ relative to the blank, calculated from nine fitted impedance spectra obtained from three independent replicates. Building on these results, Fig 8 illustrates the quantitative relationship between tetracycline concentration and the charge-transfer resistance R_ct_ by plotting the extracted R_ct_ values, with standard deviation error bars, against the logarithm of TET concentration (logC). The data reveal a highly linear inverse correlation, described by the regression equation:

**Table 1:** Fitted impedance parameters and analytical metrics of the tetracycline biosensor at various concentrations, including R_ct_, ΔR_ct_ relative to the blank, χ², SNR, and CV, expressed as mean ± SD (n = 9, three replicates).

C (nM)	logC	χ^2^ (×10^−4^)	R_ct_ 1 (Ω)	R_ct_ 2 (Ω)	R_ct_ 3 (Ω)	R_ct_ (Ω)	ΔR_ct_ ± SD (Ω)	SNR	CV (%)
0		3.15	1117.7	1149.7	1089.7	1119 ± 30		37	2.7
62.5	−7.2	3.39	6080.7	5350.0	5929.3	5788 ± 386	4668 ± 386	15	6.7
125	−6.9	3.29	5330.3	4724.7	4897.0	4984 ± 312	3864 ± 312	16	6.3
250	−6.6	3.06	4295.3	4538.3	4324.0	4386 ± 133	3267 ± 133	33	3.0
500	−6.3	4.57	3861	3939	4015	3938 ± 77	2819 ± 77	51	2.0
1000	−6	3.39	2927	2891	3079	2966 ± 100	1847 ± 100	30	3.4

$${\text{R}}_{\text{ct}}=\;-\text{2221}.\text{6}\times x\;-\text{ 10255},$$ (1)

where R_ct_ is expressed in ohms and *x* denotes the logarithm of tetracycline concentration. The coefficient of determination (R^2^ = 0.9865) confirms the linearity and reproducibility of the sensor response. The systematic decrease in R_ct_ with increasing tetracycline concentration indicates that electron-transfer resistance is strongly modulated by analyte concentration, underscoring the high sensitivity and analytical reliability of the proposed biosensor in the nanomolar range. Each data point represents the mean of nine measurements obtained from three independently fabricated biosensors (three measurements per device), which, although prepared at different times, displayed highly consistent responses, confirming the reproducibility and stability of the proposed design.

Using ΔR_ct_ relative to the blank and a linear calibration versus concentration (125–1000 nM), the limit of detection (LOD) and limit of qualification (LOQ) were calculated as 42 nM and 138 nM, respectively, based on 3σ and 10σ of the blank (see the detailed calculations in S1 Appendix). This low detection limit highlights the sensor’s high sensitivity, which can be attributed to the synergistic interplay between the CPE-R_ct_ configuration in the equivalent circuit and the optimized nanostructured electrode surface.

**Fig 9:**
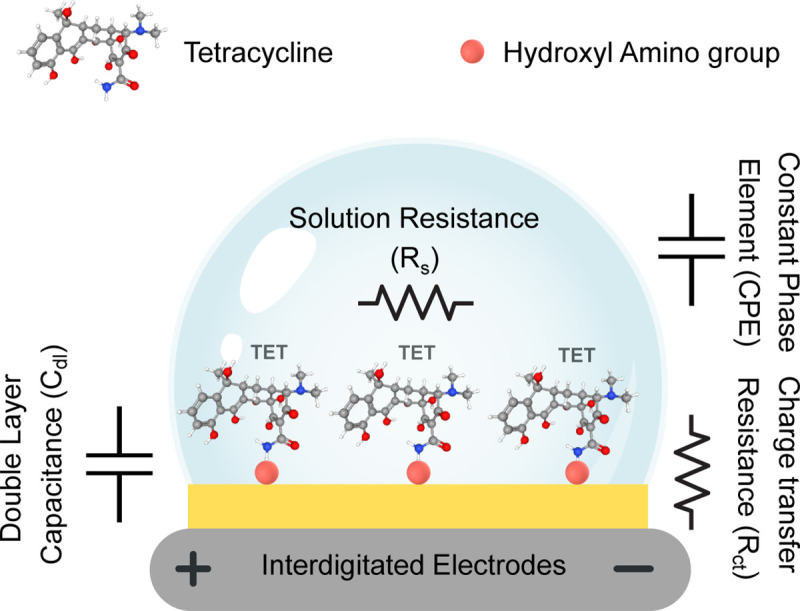
Schematic illustration of the proposed biosensor interface and its interaction with tetracycline molecules.

The underlying sensing mechanism originates from a charge-transfer process that is highly sensitive to tetracycline adsorption at the electrode surface, as shown in Fig 9. As TET molecules bind to the active sites, the resulting modulation of interfacial electron transfer leads to a progressive decrease in R_ct_, corresponding to higher current response and more efficient charge exchange at the electrode surface. This behavior might be due to TET molecules interacting with the functionalized material layer through hydroxyl and amino groups (-COOH and -NH_2_), forming a stable and orderly molecular arrangement on the sensor surface [16]. Such molecular organization enhances electron transfer from tetracycline molecules to the electrode interface, thereby facilitating charge transport, increasing surface current, and reducing interfacial resistance R_ct_. Furthermore, the strong linear correlation (R^2^ = 0.9865) between the experimental data and the linear regression model reinforces the biosensor’s high reliability, sensitivity, and analytical precision, confirming that the electron-transfer process is systematically modulated by TET concentration.

**Fig 10:**
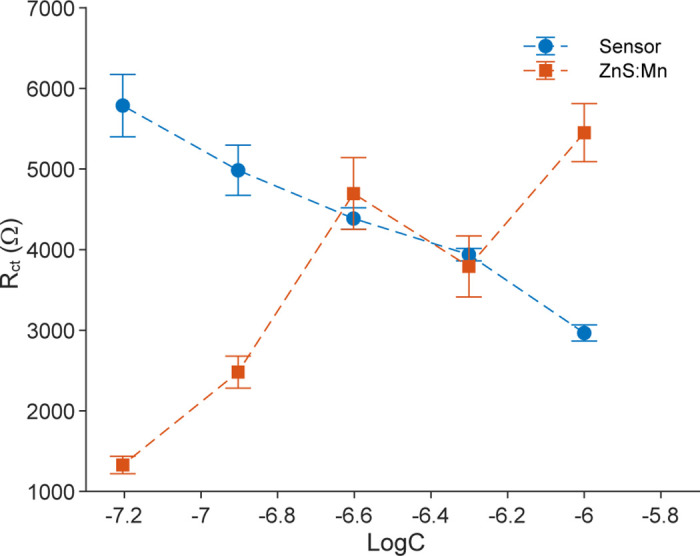
Change in charge-transfer resistance R_ct_ as a function of the logarithm of TET concentration for the proposed Mn:ZnS-CH biosensor and the Mn:ZnS-based sensor in DI water. Error bars represent the standard deviations of three replicate measurements.

The mechanistic role of chitosan was further elucidated by comparing the Mn:ZnS-CH sensor with an Mn:ZnS sensor without chitosan (Fig 10). The Nyquist plots of the Mn:ZnS-based biosensors at different tetracycline concentrations are shown in S2 Fig. Notably, the two systems exhibited opposite electrochemical trends: for the chitosan-capped nanocomposite, R_ct_ decreased with increasing tetracycline concentration, whereas in the chitosan-free Mn:ZnS system, R_ct_ increased under the same conditions. This inverse behavior indicates that fundamentally different interfacial mechanisms govern analyte interaction and electron transfer in the two systems.

In the chitosan-free Mn:ZnS system, the surface is relatively inorganic, rigid, and weakly functionalized. TET molecules interact primarily through non-specific adsorption and weak coordination with exposed Zn^2+^/Mn^2+^ surface sites. As TET concentration increases, adsorbed TET molecules progressively block active sites and introduce charge-trapping and surface passivation effects. In addition, changes in local surface polarity and the formation of a partially insulating organic layer further hinder charge transport, resulting in a concentration-dependent increase in R_ct_.

In contrast, the chitosan matrix provides a soft, hydrophilic, and functional interfacial layer rich in functional groups (-NH_2_ and -OH). These functionalities facilitate hydrogen bonding and electrostatic interactions with TET, promoting preferential and more ordered adsorption at the interface. Such interactions modulate the interfacial charge distribution, improve ion accessibility, and facilitate charge redistribution within the chitosan matrix, leading to enhanced interfacial charge-transfer kinetics and the experimentally observed decrease in R_ct_ with increasing TET concentration. Rather than acting as an electron-blocking layer, TET binding in this system dynamically modifies the interfacial environment to lower the effective charge-transfer barrier. The opposite R_ct_ trends therefore reflect two competing interfacial regimes: (i) Mn:ZnS without chitosan-TET adsorption, dominated by surface blocking and charge trapping, resulting in increased R_ct_; and (ii) Mn:ZnS-CH-TET–chitosan interactions that facilitate interfacial charge transport, resulting in decreased R_ct_.

It should be noted that the proposed analyte-sensor interaction mechanism is inferred rather than quantitatively verified in the present study. No adsorption isotherms, binding affinity constants, or post-adsorption spectroscopic analyses (e.g., FTIR or XPS) were performed to directly quantify TET-chitosan interactions. Within the scope of this work, the mechanism is supported by the consistent, concentration-dependent EIS response and well-established physicochemical considerations reported in prior literature. These findings indicate that chitosan functions not only as a stabilizing scaffold but also as an active electrochemical mediator, enhancing both the sensitivity and selectivity of the EIS-based sensor. This role is fundamentally different from the fluorescence-enhancement effect of chitosan reported in earlier optical sensing studies [15–17]. A quantitative, molecular-level elucidation of the analyte–sensor interaction, including adsorption thermodynamics, determination of binding affinity, and in situ or post-binding spectroscopic validation, will be the focus of our future work.

Table 2 summarizes the calibration curve validation results, conducted to evaluate the predictive accuracy and reliability of the developed electrochemical biosensor for tetracycline quantification. In this validation test, solutions with known tetracycline concentrations (spiked C) were analyzed using the same impedance measurement and fitting procedures established during the initial calibration. For each spiked sample, the experimentally obtained R_ct_ values were substituted into the regression equation (Eq. 1) to back-calculate the logarithm of the tetracycline concentration (logC). The calculated concentrations showed excellent agreement with the known (spiked) values across all three test points: 100 nM, 200 nM, and 450 nM. The deviations between the calculated and actual logC values were remarkably low, at 0.2%, 2.35%, and 0.76%, respectively. These low errors demonstrate the robustness of the calibration model and its strong predictive capability within the investigated concentration range. The small differences between measured and estimated values further confirm that the linear relationship between R_ct_ and log C is reproducible and stable, even under minor variations in measurement conditions.

**Table 2:** Validation of the calibration curve.

Spiked C (nM)	Spiked logC	Fitted R_ct_ (Ω)	Calculated logC	Difference (%)
100	−7	5264 ± 324	−6.99	0.2%
200	−6.7	4979 ± 415	−6.86	2.35%
450	−6.35	3960 ± 131	−6.40	0.76%

The minimal error margins indicate that the sensor’s analytical performance is only weakly affected by experimental noise or small environmental fluctuations, supporting consistent results during repeated measurements. This successful validation shows that the regression model can be confidently applied to unknown samples, providing precise quantitative results for practical analyses. Moreover, using impedance-derived R_ct_ as the quantitative transduction parameter enables rapid, label-free, and reproducible detection, positioning the biosensor as a promising analytical platform for antibiotic monitoring in food safety and environmental applications.

Overall, the results in Table 2 confirm that the established calibration model provides highly accurate estimates of tetracycline concentration, reinforcing the reliability, precision, and practical applicability of the proposed biosensor for quantitative electrochemical detection in real aquaculture monitoring scenarios.

### The selectivity of the proposed biosensors

Selectivity is a critical attribute for the practical application of biosensors, as it determines their ability to recognize the target analyte in the presence of potentially interfering substances. In this study, the selectivity of the Mn:ZnS-CH-based biosensor was systematically evaluated against six commonly used antibiotics: TET, ampicillin (AMP), amoxicillin (AMX), cephalexin (CEX), doxycycline (DOX), and penicillin (PEN), and a non-antibiotic, glucose. S3 Fig shows the Nyquist plots obtained when the biosensor was exposed to each antibiotic at a concentration of 62.5 nM. The blue symbols represent the experimental impedance spectra, while the solid red lines correspond to the Randles circuit fits. The markedly different semicircle diameters, and thus charge-transfer resistances R_ct_, indicate that the sensor responds differently to each antibiotic.

**Fig 11:**
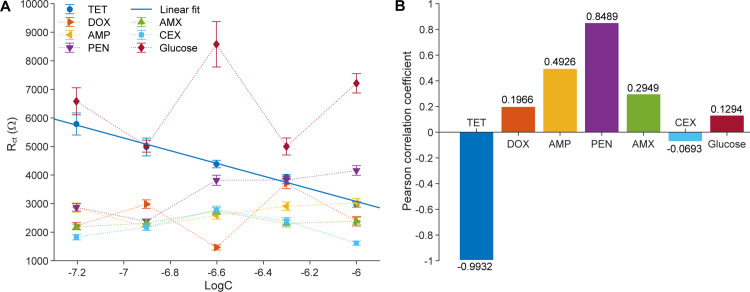
Selectivity of the proposed sensor. **(A)** Change in charge-transfer resistance R_ct_ as a function of the logarithm of concentration logC for the proposed biosensor exposed to different antibiotics. **(B)** Corresponding linear correlation coefficients between R_ct_ and logC for each antibiotic.

Fig 11A presents the change in R_ct_ as a function of the logarithm of antibiotic concentration (logC) for all seven analytes, and Fig 7B summarizes the corresponding linear correlation coefficients (Pearson correlation coefficient) between R_ct_ and logC. For tetracycline, the biosensor exhibits a clear downward linear trend, with R_ct_ decreasing strongly as TET concentration increases. In contrast, the other antibiotics yield scattered data with weak or negligible correlation between R_ct_ and logC, their correlation coefficients range only from −0.0693 to 0.8489. These results indicate that AMP, AMX, CEX, DOX, PEN, and glucose do not induce systematic or significant impedance changes at the electrode surface within the studied concentration range. The detailed linear regression equations for the proposed sensors, using different analytes, are shown in Table 3.

**Table 3:** Linear regression equations for the proposed sensors at different analyte concentrations.

Medium	Calibration equation	R^2^	Norm of residuals
TET	y = −2221.6 × *x* – 10255	0.9865	254.4
DOX	y = 348.4x + 4851	0.0387	1654
AMP	y = 350.8x + 5030	0.2427	589.9
PEN	y = 1338x + 12240	0.7207	792.9
AMX	y = 130.8x + 3249	0.0869	403.4
CEX	y = −67.32x + 1717	0.0048	922.1
Glucose	y = 414.5x + 9213	0.0168	3023

The clear separation between tetracycline and non-tetracycline responses in Fig 11 highlights the strong chemical selectivity of the biosensor. The steep negative slope observed for TET reflects an efficient interfacial electron-transfer process triggered by specific binding interactions between tetracycline molecules and the Mn:ZnS-CH functional layer. By contrast, the nearly horizontal or irregular trends for the other antibiotics confirm the absence of comparable adsorption or charge-transfer modulation.

Taken together, these observations demonstrate the exceptional selectivity of the proposed biosensor. The unique molecular compatibility between tetracycline’s hydroxyl (-OH) and amino (-NH_2_) groups and the chitosan-based nanocomposite surface enables strong hydrogen-bonding and electrostatic interactions, which facilitate charge exchange and generate a pronounced impedance response. Structurally different antibiotics lack the same combination or arrangement of complementary functional groups, leading to only minor variations in R_ct_. This high specificity ensures accurate, interference-free detection of tetracycline in complex matrices such as aquaculture water, underscoring the biosensor’s suitability for real-world antibiotic monitoring and food-safety applications.

### Stability of the proposed sensors

The temporal stability of the proposed impedimetric sensor was systematically investigated to evaluate both the storage stability of the sensing material and the signal drift during repeated measurements. A single batch of the sensing nanocomposite was prepared and stored in a sealed bottle under ambient conditions for two months. This stored material was subsequently used to fabricate multiple sensors following the same coating and fabrication protocol, ensuring consistency in sensor preparation.

To assess short- and intermediate-term stability, three independently fabricated sensors were exposed to a fixed tetracycline concentration of 250 nM at different time points after sensor preparation, including immediately after fabrication (0 min), 30 min, 60 min, 90 min, 120 min, 1 day, 3 days, 1 week, and 2 weeks. For each sensor, three consecutive electrochemical impedance spectroscopy measurements were recorded, yielding a total of nine measurements per time point. The average charge-transfer resistance, corresponding standard deviation (SD), and relative change in R_ct_ were obtained from fitted Nyquist plots and are summarized in Table 4.

**Table 4:** Fitted charge-transfer resistance R_ct_ of sensors exposed to 250 nM TET at different time points.

Time	R_ct_-AVE (Ω)	SD(Ω)	ΔR_ct_ (%)
0 min	4258.11	192.62	
30 min	4082.89	199.10	4.12
60 min	4115.56	276.66	3.35
90 min	4466.33	121.62	4.89
120 min	4321.00	183.76	1.48
1 day	4159.22	269.83	2.32
3 days	4426.11	196.31	3.95
1 week	4596.22	207.30	7.94
2 week	4347.78	259.96	2.11

To quantify temporal signal drift, the relative change in R_ct_ with respect to the initial value R_ct,0_ (t = 0) was calculated using the following expression:


$$\Delta R_{ct}=\frac{R_{ct}-R_{ct,\text{0}}}{R_{ct,\text{0}}}\times \text{100}$$ (2)

As shown in Table 4, all relative variations in R_ct_ remained below 8% throughout the entire monitoring period. Notably, within the first 24 h, the relative change was consistently below 5%, indicating that short-term signal drift is negligible and that the sensor response is highly stable over typical measurement timescales. Even at extended time points of one and two weeks, only minor fluctuations in R_ct_ were observed, with no systematic increasing or decreasing trend, suggesting the absence of progressive electrode degradation, sensing layer detachment, or loss of interfacial integrity.

Despite the use of independently fabricated sensors and repeated measurements, the fitted R_ct_ values exhibited good consistency, demonstrating excellent reproducibility of the sensor fabrication process. Moreover, comparison with the baseline R_ct_ values reported in Table 1 for sensors fabricated using the same material batch after two months of storage (4386 ± 133 Ω) revealed close agreement with the initial R_ct_ obtained in this stability study (4258 ± 192 Ω). This observation confirms that prolonged storage of the sensing material does not adversely affect the electrochemical properties or sensing performance of the fabricated electrodes.

Although the present investigation was limited to a two-week evaluation period, the consistently low signal variation and stable impedance response across all time points provide strong evidence of the long-term stability of the proposed sensor. The absence of measurable performance degradation, combined with the demonstrated material storage stability, highlights the robustness and reliability of the sensing platform. In future work, extended stability studies over longer durations will be conducted to further assess the operational lifetime and storage durability of the sensors under various environmental and handling conditions.

### Applications of proposed biosensors to detect TET in different working media

**Fig 12:**
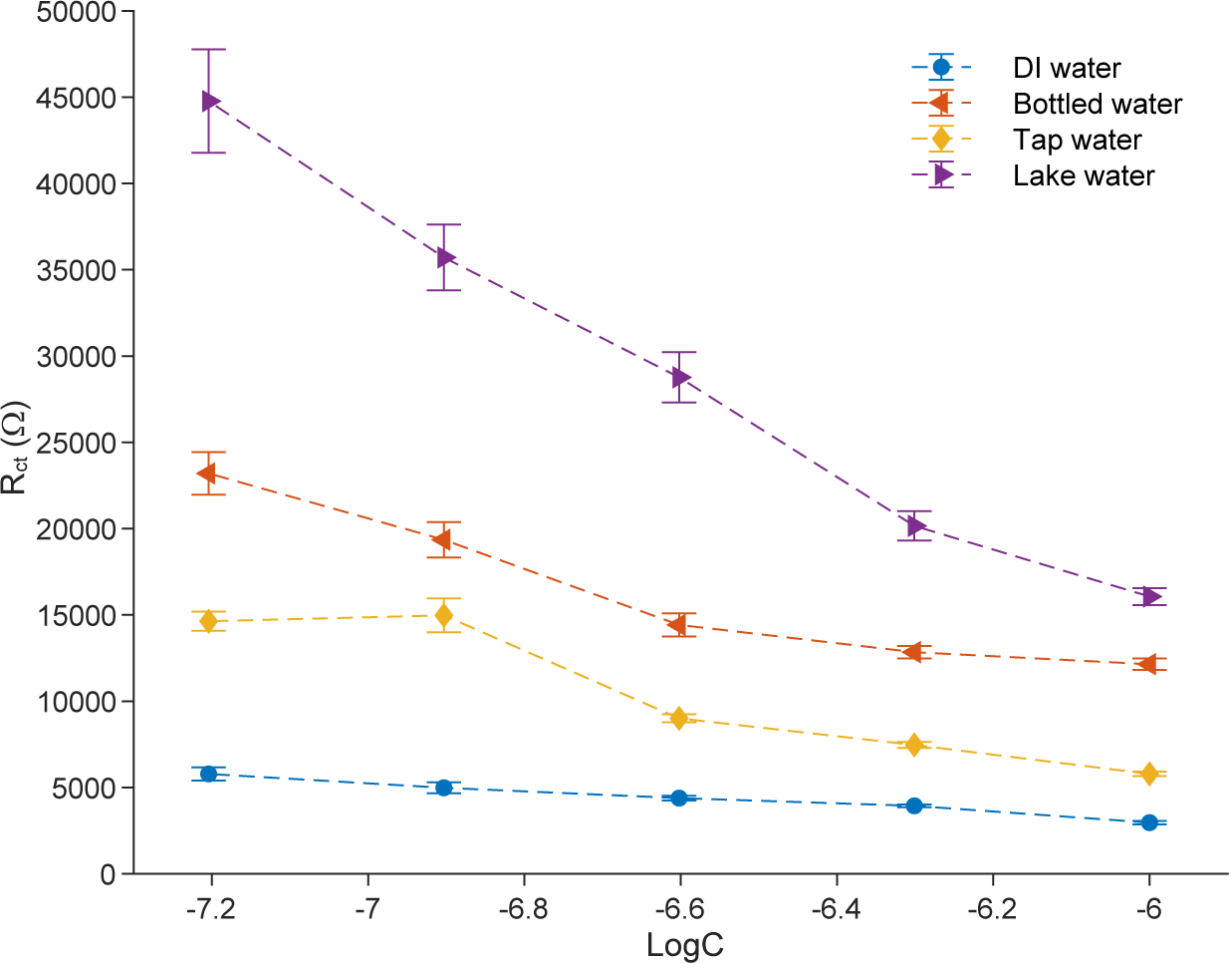
The change in R_ct_ values with logC when the proposed sensors are exposed to TET in different water matrices.

To assess the applicability of the Mn:ZnS-CH impedimetric biosensor for real aquaculture monitoring, its performance was evaluated in several representative media, including bottled water, tap water, and lake water. The Nyquist plots obtained at 62.5 nM tetracycline in each medium are shown in S4 Fig. Across the full concentration range tested, the sensor response, expressed as the charge-transfer resistance R_ct_, decreased consistently with increasing tetracycline concentration in all media (Fig 12), confirming that the biosensor can reliably detect tetracycline under diverse conditions.

The linear regression parameters extracted from the calibration curves in each medium are summarized in Table 5. All media exhibit good linearity, with coefficients of determination (R²) ranging from 0.8987 to 0.9894, indicating stable and predictable sensor behavior across different matrices. Sensor sensitivity, represented by the slope of the calibration equation, shows a moderate dependence on the sample matrix. The highest sensitivity was observed in lake water (slope = −24238), followed by bottled water (−9509), tap water (−8373), and DI water (−2222).

**Table 5:** Linear calibration parameters of the proposed biosensor for tetracycline detection in different media.

Medium	Calibration equation	R^2^
DI	y = −2222*x* – 10255	0.9865
Bottled water	y = -9509*x* − 46390	0.9139
Tap water	y = -8373*x* − 44900	0.8987
Lake water	y = -24238*x* − 130900	0.9873

y – change in charge-transfer resistance Rct (Ω); *x* – logarithm of tetracycline concentration.

Although a detailed chemical characterization of the water matrices (e.g., organic matter content, ionic composition, or conductivity) was not performed in this study, the enhanced sensitivity observed in lake water can be plausibly attributed to the combined presence of naturally occurring ions and natural organic matter. These matrix constituents are known to increase ionic strength, modify the electrical double layer, alter interfacial charge-transfer kinetics, and influence TET speciation or complexation, thereby amplifying the fitted R_ct_ response and the corresponding calibration slope. Such matrix-induced enhancement has been widely reported for impedance-based sensors operating in natural waters and reflects interfacial modulation rather than sensor instability. In contrast, measurements in DI, bottled, and tap water produced lower slopes while maintaining excellent linearity and minimal deviation, demonstrating that the biosensor operates reliably even in less complex matrices without the need for sample pretreatment or dilution.

The observed matrix-dependent sensitivity is further consistent with the influence of pH variation across the tested environments. The measured pH values were 5.94 for DI water, 7.93 for tap water, 7.94 for bottled water, and 7.92 for lake water, spanning a mildly acidic to near-neutral range relevant to aquaculture systems. DI water, with a slightly acidic pH, exhibited the lowest sensitivity. At lower pH, partial protonation of chitosan amino groups (-NH_2_ → -NH_3_⁺) can weaken hydrogen bonding and electrostatic interactions with TET molecules and alter the electrical double layer at the electrode–electrolyte interface, resulting in reduced modulation of Rct per decade of concentration. In contrast, near-neutral pH conditions favor balanced protonation of chitosan functional groups and stable TET speciation, enhancing analyte–surface interactions and interfacial charge redistribution, which leads to higher sensitivity.

Importantly, the particularly high sensitivity observed in lake water cannot be attributed to pH alone, as its pH is comparable to that of tap and bottled water, but rather reflects the combined influence of near-neutral pH with additional matrix constituents such as ions and natural organic matter. Overall, these results indicate that pH variations within the typical aquaculture range (approximately pH 6–8) modulate sensor sensitivity without compromising linearity, operational stability, or detection capability. Taken together, the consistent linear response, tolerance to matrix effects, and stable performance across realistic pH conditions underscore the suitability of the Mn:ZnS-CH/IDE impedimetric biosensor for direct, on-site monitoring of tetracycline residues in aquaculture and natural water environments.

The analytical performance of the Mn:ZnS-CH/IDE impedimetric biosensor was also benchmarked against recent tetracycline detection platforms. For example, nanodiamond-starch sensors have achieved detection in the 5–180 micromolar range with a LOD of about 2 μM [25], while aptamer-gold nanoparticle sensors have reached sub-nanomolar LODs around 0.12 nM [26]. Molecularly imprinted polymer (MIP) sensors based on ZnO have reported very low LODs (approximately 0.02 nM) but require complex synthesis and lengthy fabrication procedures [27]. Compared with these systems, the present biosensor offers a balanced compromise between analytical performance and practical implementation. Although its LOD (42 nM) is higher than that of aptameric or MIP-based platforms, the proposed system is enzyme-free, label-free, low-cost, biodegradable, and disposable, which are key features for scalable field deployment. In addition, the impedance-based transduction enables real-time, label-free quantification without extra reagents or biological recognition elements, simplifying both operation and maintenance.

In our previous work, we employed Mn:ZnS-chitosan nanocomposites to achieve picomolar-level detection of TET using a fluorescence-based optical approach [16]. While optical sensors can deliver outstanding sensitivity, integrating them into compact, rugged, and easy-to-use devices for field applications is generally more challenging than for electrochemical systems. By integrating Mn:ZnS nanostructures with a chitosan biopolymer matrix on interdigitated electrodes, the developed biosensor achieves both high selectivity and operational stability across multiple real-world samples. The findings validate its feasibility for rapid and routine monitoring of antibiotic residues in aquaculture water and food products. Future efforts will aim to further reduce the detection limit through the optimization of nanomaterials and signal amplification techniques. Additionally, incorporating this sensing system into portable readout devices could enable on-site, multiplexed detection of various antibiotics or contaminants, thereby enhancing its utility in environmental surveillance and public health applications.

In summary, the Mn:ZnS-CH/IDE impedimetric biosensor demonstrated consistent, linear, and selective detection of tetracycline in different aqueous and dairy media, confirming its robustness and tolerance to matrix effects. Comparative experiments with the chitosan-free Mn:ZnS sensor showed that chitosan plays a decisive role in facilitating electron transfer and molecular recognition. Together, these results highlight the strong potential of this eco-friendly, disposable biosensor platform for real-sample antibiotic monitoring and lay the foundation for the development of portable, field-deployable analytical systems.

## Limitations and future perspectives

While the proposed Mn:ZnS-CH/IDE impedimetric biosensor demonstrates reliable, sensitive, and selective detection of tetracycline under controlled laboratory conditions, several limitations should be acknowledged to clearly define the scope of the present study and to guide future research directions.

First, physicochemical characterization of the Mn:ZnS-CH nanocomposite synthesized in this work was performed using XRD, SEM, EDX, and FTIR to confirm crystallinity, elemental composition, morphology, and functional groups. However, quantitative surface roughness analysis (e.g., AFM or profilometry) and direct post-binding spectroscopic evidence (such as FTIR band shifts, XPS, or UV-Vis measurements after tetracycline adsorption) were not conducted. Consequently, the proposed interaction mechanisms between tetracycline and the chitosan-modified interface are inferred from electrochemical behavior and established literature, rather than being directly verified. Future studies will incorporate surface-sensitive and spectroscopic techniques to quantitatively correlate morphology, roughness, and molecular-level interactions with impedance responses.

Second, the equivalent circuit model was selected based on physical relevance, fitting stability, and goodness-of-fit within the experimentally accessible frequency window (4–10^4^ Hz). Very low-frequency diffusion processes (< 4 Hz) could not be probed due to instrumental limitations, and Warburg-type elements were therefore not explicitly validated. Although a constant phase element CPE was included to account for non-ideal interfacial capacitance, CPE-related parameters were not employed as analytical outputs because of their sensitivity to secondary interfacial effects. Extended frequency measurements using potentiostats with sub-Hz capability, together with broader model comparisons, may further refine mechanistic interpretation in future work.

Third, stability and reproducibility assessments focused on operational and short-to-intermediate-term performance. The sensor exhibited minimal R_ct_ drift (≤ 8%) over repeated measurements and storage periods of up to two weeks, confirming robustness for single-use deployment. Nevertheless, long-term shelf-life, controlled-humidity storage, and inter-laboratory reproducibility were not systematically evaluated. These aspects will be addressed in future studies aimed at practical and large-scale deployment.

Fourth, real-sample validation was performed using spiked water matrices (DI, tap, bottled, and lake water) to enable controlled recovery analysis. Naturally contaminated aquaculture samples containing unknown tetracycline residues were not tested, as independent reference methods (e.g., LC-MS/MS) were not available for quantitative validation. Future work will focus on testing authentic aquaculture samples and benchmarking sensor performance against established analytical techniques.

Fifth, the device was intentionally designed for low-cost, disposable, single-use operation. Systematic regeneration or multi-cycle reuse studies were not pursued due to irreversible or partially reversible analyte adsorption, polymer hydration effects, and potential fouling in complex matrices. Although preliminary observations suggest baseline drift upon reuse, comprehensive regeneration strategies were beyond the scope of the present study and represent a potential direction for future investigation.

Building on the present proof-of-concept, several complementary research directions will be pursued to advance the Mn:ZnS-CH/IDE impedimetric biosensor toward practical deployment. Future efforts will focus on integrating the IDE-based sensor with miniaturized, low-power, and wireless impedance readout electronics, such as Bluetooth- or NFC-enabled modules, to enable real-time, on-site monitoring without reliance on bulky laboratory instrumentation. In parallel, incorporation of microfluidic platforms will be explored to improve sample handling, reduce required sample volume, control flow conditions, and enhance measurement reproducibility. Microfluidic integration is also expected to facilitate automated calibration, multiplexed sensing, and mitigation of matrix effects commonly encountered in complex water samples.

Further optimization will address sensor performance under variable environmental conditions relevant to aquaculture systems, including temperature fluctuations, pH variation, ionic strength, and biofouling-prone matrices, to ensure reliable operation under realistic field conditions. In addition, the sensing platform will be extended toward expanded analytical capability by modifying the sensing layer or electrode architecture to enable simultaneous detection of multiple antibiotics or environmental contaminants. Collectively, these developments are essential to translate the proposed Mn:ZnS-CH/IDE biosensor from laboratory validation into a scalable, robust, and field-deployable monitoring system for environmental and aquaculture applications.

## Conclusion

In this work, we report for the first time a non-enzymatic impedimetric biosensor that integrates Mn-doped ZnS–chitosan nanocomposites with interdigitated electrodes for tetracycline detection. In contrast to previous optical platforms using similar nanomaterials, this device exploits modulation of electrochemical charge transfer at the biopolymer–nanocomposite interface, providing a distinct sensing mechanism and enabling direct, label-free quantification in real samples. The biosensor exhibits a strong linear response from 62.5 nM to 1000 nM, together with high reproducibility and operational stability across different water matrices, confirming its suitability for field-level monitoring. It also shows pronounced selectivity for tetracycline over structurally related antibiotics such as ampicillin, amoxicillin, cephalexin, doxycycline, and penicillin, with minimal cross-reactivity. These results demonstrate that the platform combines robustness, simplicity, and analytical accuracy, making it highly relevant for applications in food safety, environmental monitoring, and pharmaceutical analysis. The successful integration of Mn-doped ZnS nanomaterials, chitosan capping, and interdigitated electrode design provides a solid basis for translating this sensing approach into portable, real-time monitoring devices. Overall, this study establishes a promising foundation for the development of advanced, eco-friendly biosensors capable of rapid, on-site detection of antibiotic residues, addressing pressing needs in public health and environmental protection.

## Supporting information

S1 Table Fitted electrochemical parameters of one representative proposed biosensor for tetracycline detection using the Randles equivalent circuit model. Each value corresponds to the mean of three repeated EIS measurements.
(PDF)

S1 Appendix Calculation of limit of detection and limit of quantification. (PDF)

S1 Fig Schematic illustration and geometric parameters of the interdigitated electrodes (IDEs). (A) The IDEs exhibit a comb-like geometry consisting of 20 fingers, with a gap spacing of 200 μm and a finger width of 400 μm. The electrodes were fabricated from aluminum coated with lead. (B) Photograph of the fabricated IDE device.
(TIF)

S2 Fig The Nyquist plots of the Mn:ZnS-based biosensors for different concentrations of tetracycline in DI water. (A) 62.5 nM, (B) 125 nM, (C) 250 nM, (D) 500 nM, and (E) 1000 nM. The blue dots represent the experimental data, while the red lines correspond to the fitted data using the Randles equivalent circuit model shown in Fig 2.
(TIF)

S3 Fig The Nyquist plots of the proposed sensors for different 62.5 nM antibiotic samples. (A) Ampicillin, (B) Amoxicillin, (C) Cephalexin, (D) Doxycycline, (E) Penicillin, (F) Glucose. The blue dots represent the experimental data, while the red lines correspond to the fitted data using the Randles equivalent circuit model shown in Fig 2.
(TIF)

S4 Fig The Nyquist plots of the proposed sensors for different concentrations of 62.5 nM tetracycline in different working media. (A) Deionized water, (B) Bottled water, (C) Tap water, (D) Lake water. The blue dots represent the experimental data, while the red lines correspond to the fitted data using the Randles equivalent circuit model shown in Fig 2.
(TIF)
