# Supplementary material for: Disposable non-enzymatic impedimetric biosensor using Mn-doped ZnS-chitosan nanocomposite for tetracycline detection
Source: PLoS One. 2026 Feb 27;21(2):e0344103. doi: 10.1371/journal.pone.0344103 (PMC12948106; doi:10.1371/journal.pone.0344103)
Supplement: S1 Table — Each value corresponds to the mean of three repeated EIS measurements. (PDF) [file pone.0344103.s001.pdf]

S1 Table. Fitted electrochemical parameters of one representative proposed biosensor for tetracycline detection using the Randles equivalent circuit model. Each value corresponds to the mean of three repeated EIS measurements.

**C (nM)**	**logC**	χ^2	**R_{ct} (Ω)**	**C_{dl} (nF)**	**CPE (μF)**	**n**	**R_s**
62.5	-7.2	3.39×10^{-4}	6080 ± 240	152 ± 3.2	1.5 ± 0.007	0.82±0.001	1029 ± 2.82
125	-6.9	3.29×10^{-4}	5330 ± 272	114 ± 3.18	1.28 ± 0.006	0.83±0.001	1089 ± 3.06
250	-6.6	3.06×10^{-4}	4295 ± 418	102 ± 6.18	2.27 ± 0.008	0.78±0.002	1172 ± 3.50
500	-6.3	4.57×10^{-4}	3860 ± 270	140 ± 5.91	2.14 ± 0.010	0.78±0.002	1060 ± 4.01
1000	-6	3.39×10^{-4}	2927 ± 247	117 ± 6.57	2.43 ± 0.004	0.78±0.002	902 ± 3.18

C: Tetracycline concentration
