# Supplementary material for: Disposable non-enzymatic impedimetric biosensor using Mn-doped ZnS-chitosan nanocomposite for tetracycline detection
Source: PLoS One. 2026 Feb 27;21(2):e0344103. doi: 10.1371/journal.pone.0344103 (PMC12948106; doi:10.1371/journal.pone.0344103)
Supplement: S1 Appendix — (PDF) [file pone.0344103.s002.pdf]

S1 Appendix. Calculation of Limit of Detection (LOD) and Limit of Quantification (LOQ)

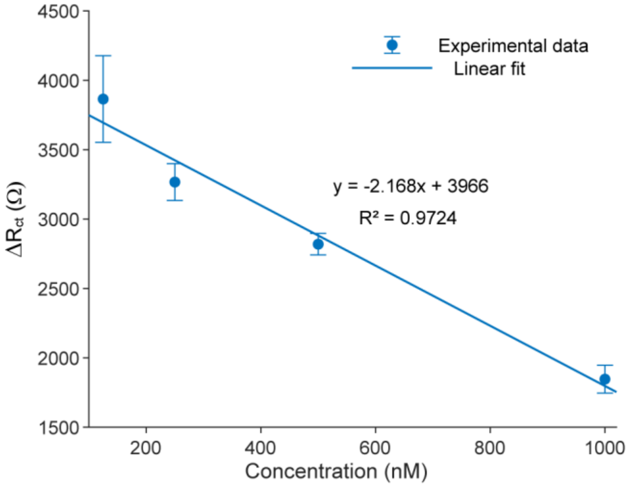

Step 1: Data:

C (nM)	ΔR_{ct}-AVE	SD
1000	1846.66	100
500	2819.21	77
250	3266.88	133
125	3864.99	312

Step 2: Fit the linear regression: ΔR_{ct} = a + b C

Calibration equation is: ΔR_{ct} = -2.1684 C + 3965.9 (R^2 = 0.972)

Step 3: Residual standard deviation of blank sample: $\sigma = 30\ \Omega$

Step 4: Calculate LOD and LOQ:

$$\text{LOD} = \frac{3\sigma}{slope} = \frac{3 \times 30}{2.168} = 42\ nM$$

$$\text{LOQ} = \frac{10\sigma}{slope} = \frac{10 \times 30}{2.168} = 138\ nM$$
