# Supplementary figures and images for: Disposable non-enzymatic impedimetric biosensor using Mn-doped ZnS-chitosan nanocomposite for tetracycline detection
Source: PLoS One. 2026 Feb 27;21(2):e0344103. doi: 10.1371/journal.pone.0344103 (PMC12948106; doi:10.1371/journal.pone.0344103)

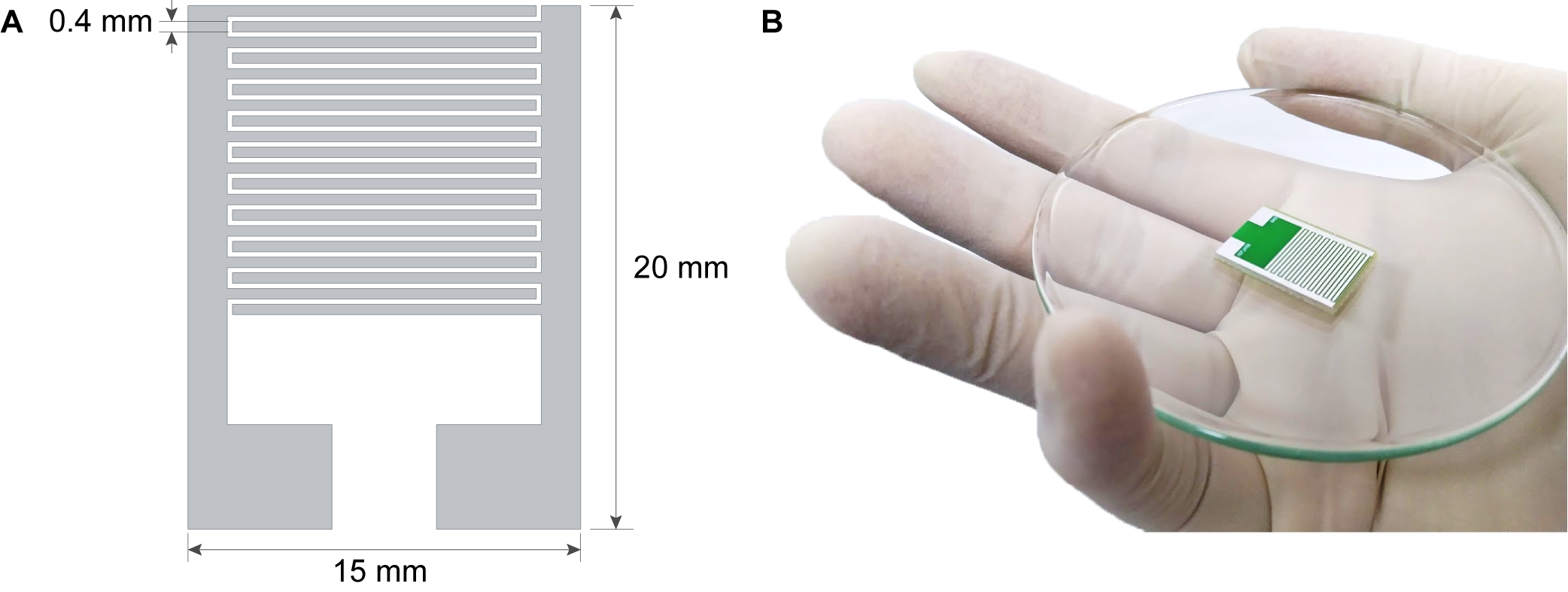

Supplement: S1 Fig — (A) The IDEs exhibit a comb-like geometry consisting of 20 fingers, with a gap spacing of 200 μm and a finger width of 400 μm. The electrodes were fabricated from aluminum coated with lead. (B) Photograph of the fabricated IDE device. (TIF) [file pone.0344103.s003.tif]

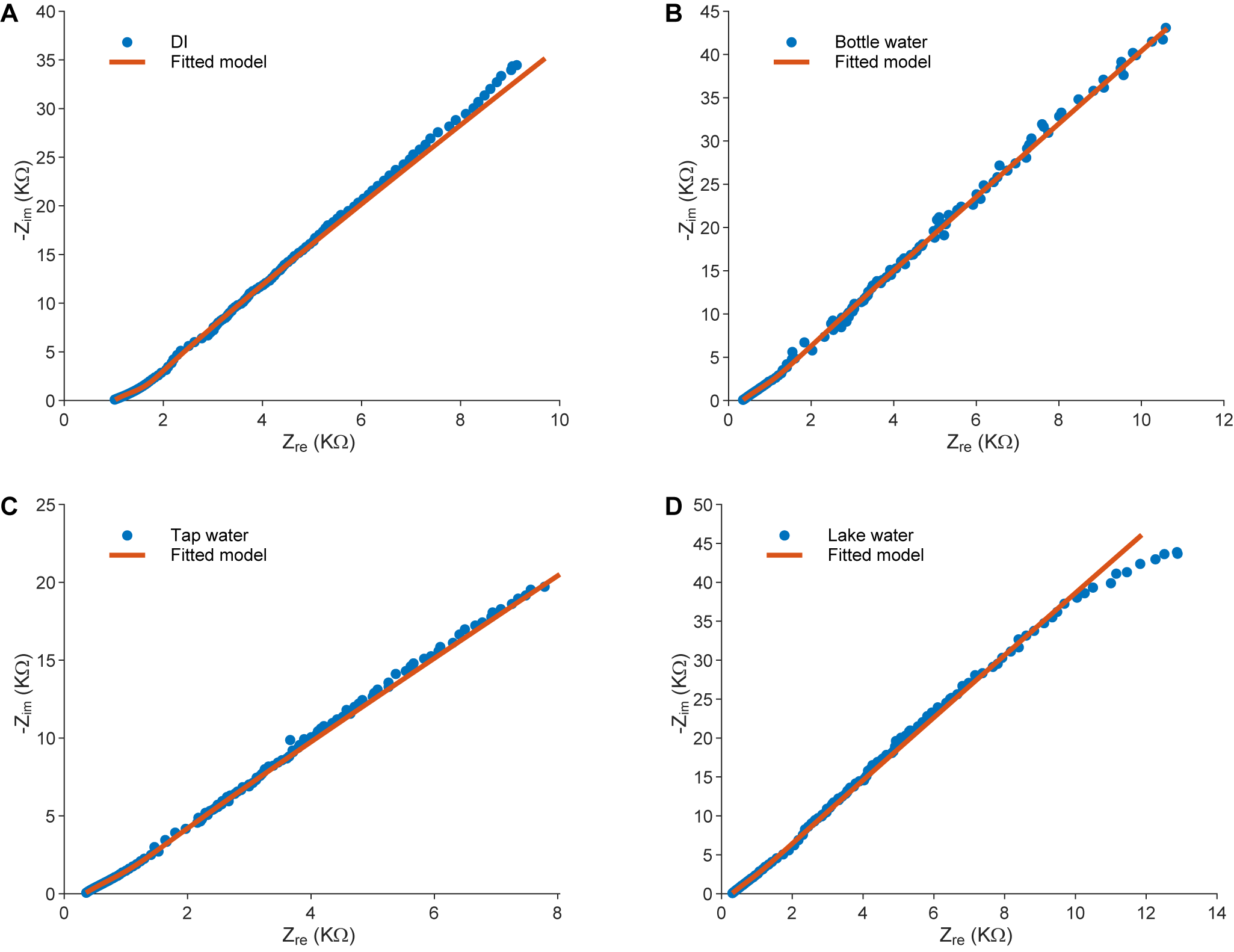

Supplement: S2 Fig — (A) 62.5 nM, (B) 125 nM, (C) 250 nM, (D) 500 nM, and (E) 1000 nM. The blue dots represent the experimental data, while the red lines correspond to the fitted data using the Randles equivalent circuit model shown in Fig 2. (TIF) [file pone.0344103.s006.tif]

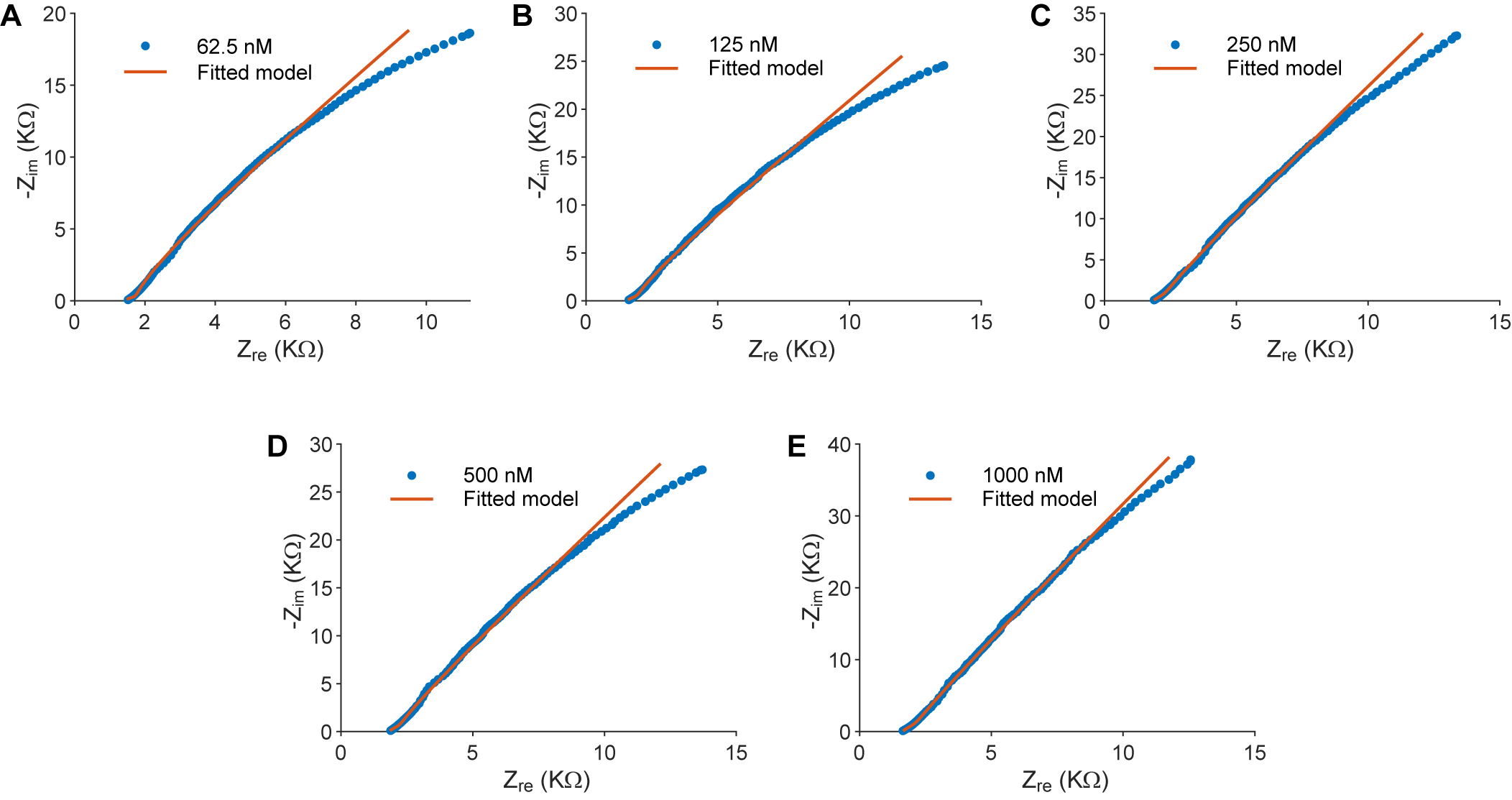

Supplement: S3 Fig — (A) Ampicillin, (B) Amoxicillin, (C) Cephalexin, (D) Doxycycline, (E) Penicillin, (F) Glucose. The blue dots represent the experimental data, while the red lines correspond to the fitted data using the Randles equivalent circuit model shown in Fig 2. (TIF) [file pone.0344103.s004.tif]

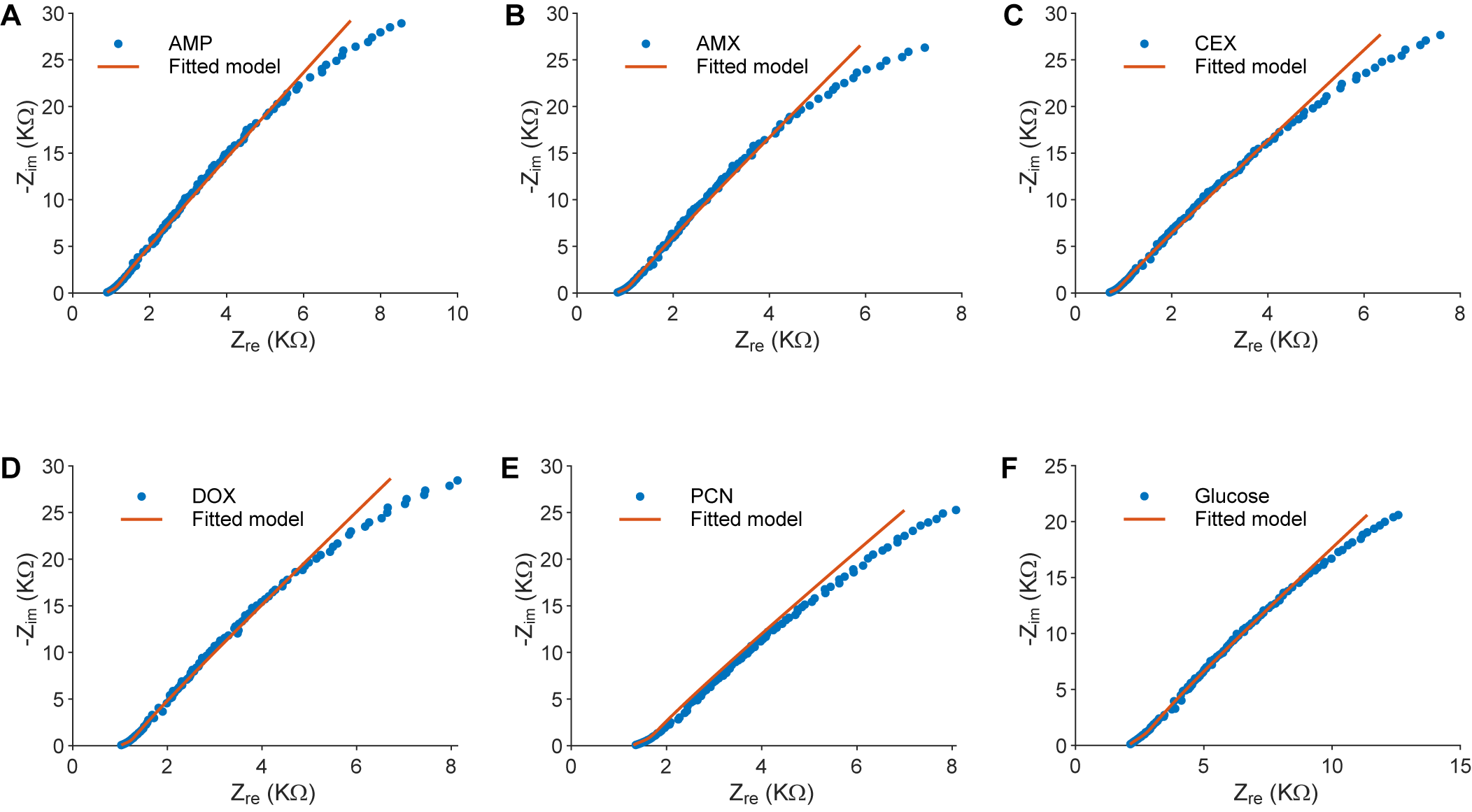

Supplement: S4 Fig — (A) Deionized water, (B) Bottled water, (C) Tap water, (D) Lake water. The blue dots represent the experimental data, while the red lines correspond to the fitted data using the Randles equivalent circuit model shown in Fig 2. (TIF) [file pone.0344103.s005.tif]
